# Heparin Assisted Photochemical Synthesis of Gold Nanoparticles and Their Performance as SERS Substrates

**DOI:** 10.3390/ijms151019239

**Published:** 2014-10-23

**Authors:** Maria del Pilar Rodríguez-Torres, Luis Armando Díaz-Torres, Sergio Romero-Servin

**Affiliations:** 1 Grupo de Espectroscopia de Materiales Avanzados y Nanoestructurados (GEMANA), Centro de Investigaciones en Óptica, A.C., Loma del Bosque 115, León, Guanajuato, C.P. 37150, Mexico; E-Mail: mprt@cio.mx; 2 Centro de Investigaciones en Óptica, A.C., Loma del Bosque 115, León, Guanajuato, C.P. 37150, Mexico; E-Mail: sromero@cio.mx

**Keywords:** heparin, gold nanoparticles, photochemistry, SERS, organic dyes

## Abstract

Reactive and pharmaceutical-grade heparins were used as biologically compatible reducing and stabilizing agents to photochemically synthesize colloidal gold nanoparticles. Aggregates and anisotropic shapes were obtained photochemically under UV black-light lamp irradiation (λ = 366 nm). Heparin-functionalized gold nanoparticles were characterized by Scanning Electron Microscopy and UV-Vis spectroscopy. The negatively charged colloids were used for the Surface Enhanced Raman Spectroscopy (SERS) analysis of differently charged analytes (dyes). Measurements of pH were taken to inspect how the acidity of the medium affects the colloid-analyte interaction. SERS spectra were taken by mixing the dyes and the colloidal solutions without further functionalization or addition of any aggregating agent.

## 1. Introduction

Spectroscopy methods based on Raman Effect are very useful techniques for chemical and physical analysis. Raman spectroscopy is complementary and sometimes competitive to other methods. It has some advantages over various electrons spectroscopies. For example, it allows following up surface processes without the requirement of high vacuum conditions. Also, Raman spectra provide more specific information about given compounds than UV and visible spectroscopy, so it makes the identification of molecules possible. Moreover, Raman spectroscopy usually requires very simple sample preparation. In spite of many advantages, the application of this method is limited by the very low intensity of normal Raman scattering. However, much more sensitive Raman techniques have been developed such as Surface Enhanced Raman spectroscopy (SERS). This method gives almost the same information on the molecules and their local interactions as normal Raman spectroscopy but ensures a great sensitivity. The Raman scattering from a compound (or ion) adsorbed on a nanostructured metal surface (or within its proximity) can significantly increase in comparison to the anayte in solution. This phenomenon was observed first time by Fleischman in 1974 and was partially explained by Jean Maire and Van Vuyne in 1977 [1].

Nanostructures of noble and transition metals onto which the molecules are adsorbed can cause enhancement of the intensity of Raman scattered light. There are two main contributions into Raman enhancement. The first is the electromagnetic (EM) enhancement related to the surface plasmon resonance (SPR). It arises when the wavelength of light couples the oscillation frequency of free conduction electrons on the metal surface. Molecules adsorbed or in close proximity to the surface are affected by relatively enormous electromagnetic field. Intensity of Raman bands depends on the orientation of the scattering molecules and an enhancement of vibrational modes normal to the surface is most efficient. The second mode of enhancement is related to short-range mechanisms of chemical nature: the charge-transfer (CT) complex formation and the chemical bonding of the adsorbate to the metallic nanoparticle surface. Earlier reports [2] focused on SERS on silver electrodes with modified roughened surfaces, but since 1979 the enhancement of Raman signal on silver and gold sols was observed in metal colloids [3]. Colloidal metal nanoparticles have allowed the testing of theoretical models of SERS phenomenon as well as opened the window to their application as an analytical tool in chemistry and biological sciences. Here, SERS efficiency of heparin assisted photochemically-synthesized colloidal gold nanoparticles was tested by using differently charged analytes: Methylene Blue (+), MB [4], Rose Bengal (−), RB [5], and Neutral Red, NR (slightly cationic with a net charge close to zero [6]), all of them at one concentration (1.996 µM).

The organic material used as stabilizing and reducing agent in the nanoparticle photochemical synthesis is heparin sodium, which is a polysaccharide that belongs to the glycosaminoglycans (GAGs) family [7]. The synthesis method, developed in an earlier work [8], is revisited and the products are applied to SERS studies. Heparin-based nanoparticles have been synthesized previously by thermal methods with a direct functionalization of the products [9,10,11], and in other cases, functionalization is performed after their synthesis [12]. In this work, black light is used to trigger heparin’s reducing action exploiting its reactivity in the UV range. This photochemical method provides a good green chemistry alternative, and in addition, its setup cost is low. Green chemistry methods for nanoparticle synthesis are being incorporated and studied due to the following advantages: Use of non-pollutant nor hazardous reactants, use of reactants that are biocompatible, the development of approaches whose production processes of nanomaterials require minimal energy consumption, and a minimal disposal of byproducts [13]. Green photochemical approaches have been explored before, using molecules such as alginate, dopamine hydrochloride, and collagen [14,15,16] or even without any agent present besides the metal precursor [17]. Also, while SERS substrates based on biomolecules like chitosan, agar, adenine, plant extracts or hybrid (CTAB and DNA) have been the subject of study for analyte detection [18,19,20,21], to our knowledge, heparin-based nanoparticles for SERS have not yet been reported.

In SERS it is important to know how the analytes interact with metal nanoparticles. Dyes such as Rhodamines (B OR 6G, commonly) [22,23] and Crystal Violet [24] have already been detected with the aid of gold nanoparticles, yielding good SERS signals, as well as Methylene Blue using plant-synthesized gold nanoparticles [25], and Rose Bengal by using citrate reduced gold colloids [26]. Neither SERS experiments on Neutral Red detection, nor heparin-based gold nanoparticle results in SERS studies have been reported to our knowledge.

In the present work, a 1 mM solution from each dye (MB, RB and NR) was prepared to measure normal Raman spectra. Then, the analyte was added to the gold nanoparticle solution (either elaborated from reactive or pharmaceutical-grade heparin) and the SERS spectra were taken. Then the effects of analyte charge and the performance of these nanoparticles for SERS are discussed.

## 2. Results and Discussion

### 2.1. UV-Vis Spectroscopy Measurements

**Figure 1:**
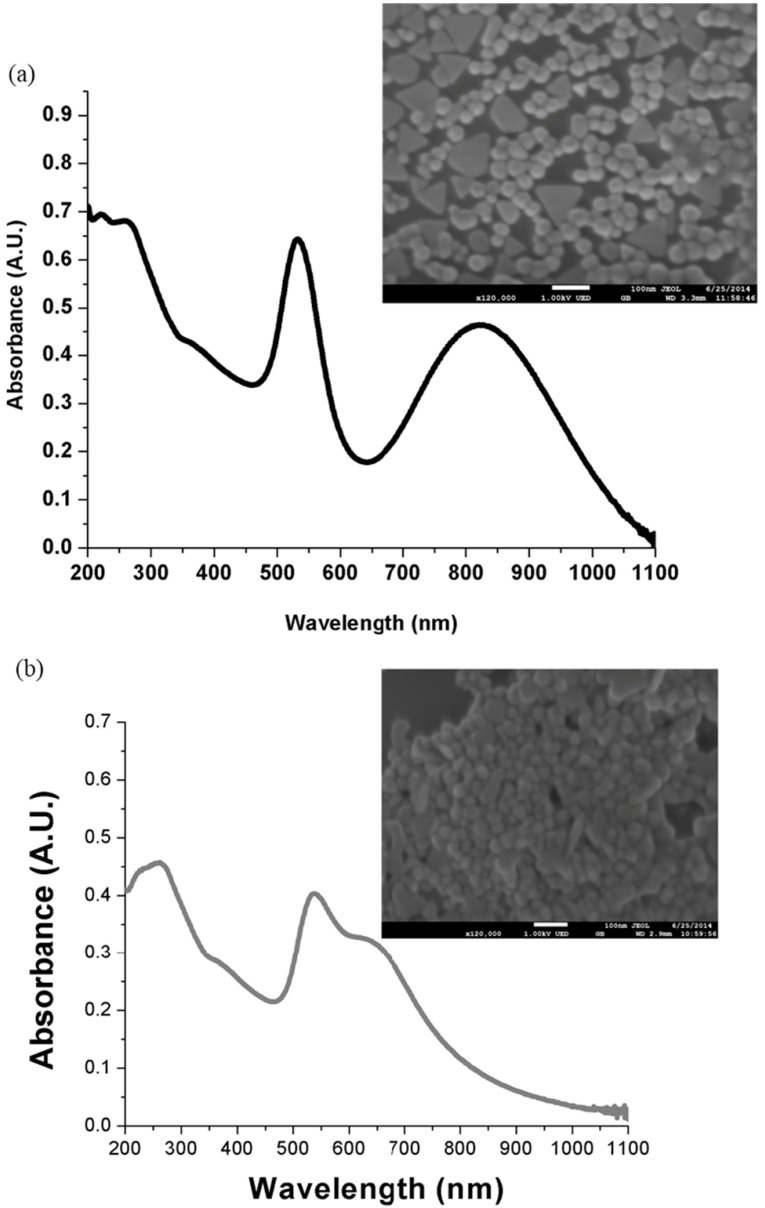
(**a**) UV-Vis spectra of pharmaceutical-grade heparin nanoparticle synthesis; (**b**) UV-Vis spectra of reactive-grade heparin nanoparticle synthesis.

UV-Vis spectra were taken to analyze the nanoparticles absorption characteristics and interaction with analytes. The pharmaceutical-grade synthesized nanoparticles show two absorption peaks, one at 532 nm and another one at 824 nm, which means that such products are anisotropic, having a longitudinal as well as a transverse mode (Figure 1a). This can be verified by the scanning electron microscope images. The SEM micrograph inset in Figure 1a shows nanoparticles with either a quasispherical shape, or triangular and trapezoidal plates. Sizes vary from 45 to 60 nm for the quasispherical products while triangles are about 45–75 nm. The situation is completely different, however, for the reactive-grade heparin nanoparticles. The UV-Vis spectra shows one band at 536 nm and a bump to its right side at 630 nm which indicates aggregation in the nanoparticles, see Figure 1b. Quasispherical particles with sizes in the 30–35 nm range are observed in SEM micrograph inset in Figure 1b. The distribution is more monodisperse than the one shown by pharmaceutical-grade heparin synthesized nanoparticles. No other morphology is observed, but it is clear that aggregates are formed.

**Figure 2:**
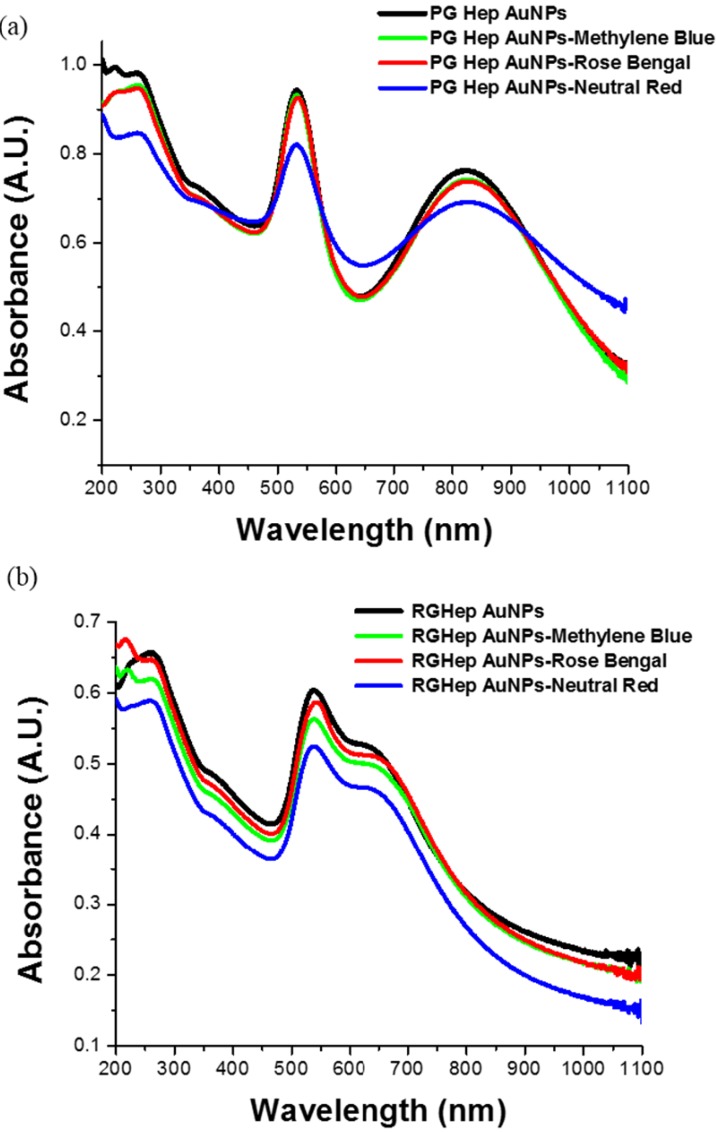
(**a**) UV-Vis spectra of pharmaceutical-grade heparin nanoparticles synthesis and its mix with the differently-charged dyes; (**b**) UV-Vis spectra of reactive-grade heparin nanoparticles synthesis and its mix with the differently-charged dyes.

UV-Vis spectra (Figure 2a,b) were measured to monitor how the nanoparticles interact with the studied analytes; however there are no dramatic changes in absorption spectra of the mixed solution of colloidal nanoparticles and dyes. In the case of the pharmaceutical-grade nanoparticles (Figure 2a), an almost negligible red-shift is observed, whereas for the reactive-grade heparin nanoparticles a small red-shift is observed (Figure 2b). Though these nanoparticles were already aggregated before analyte addition, analyte addition was found to result in an almost negligible change in absorption spectra profile. So it is not possible to discern form absorption spectra the degree of interaction between the nanoparticles and dyes.

### 2.2. Raman Measurements

**Figure 3:**
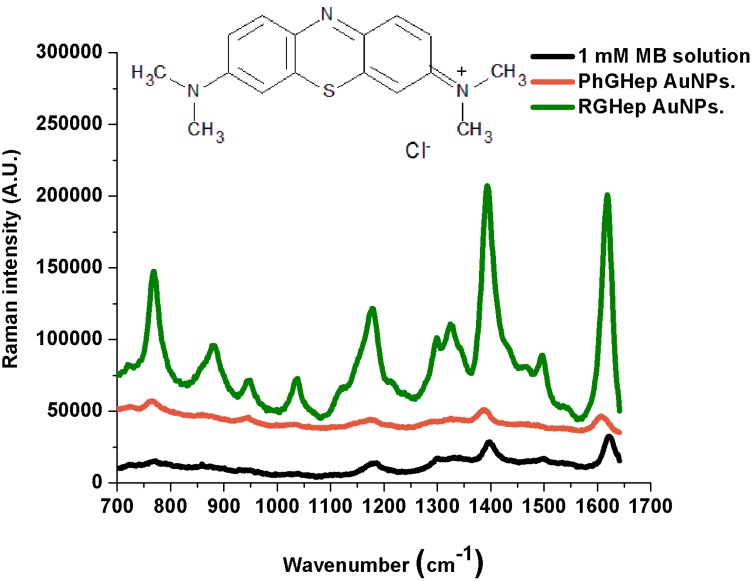
Normal Raman and SERS spectra of Methylene Blue.

**Figure 4:**
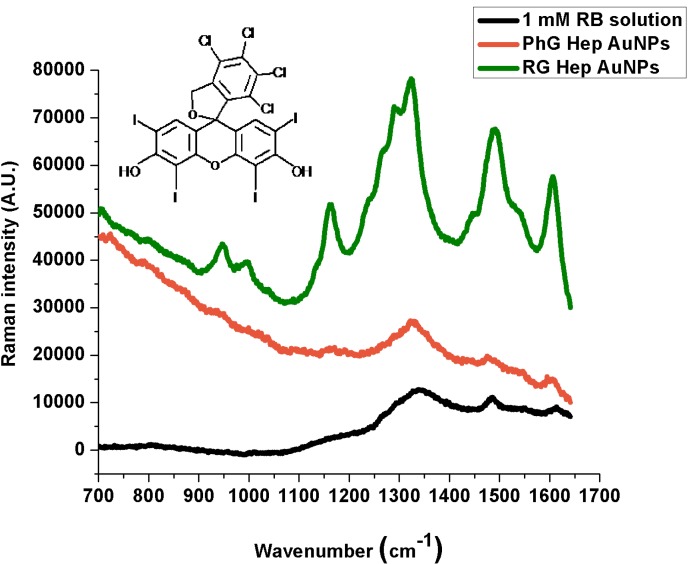
Normal Raman and SERS spectra of Rose Bengal.

**Figure 5:**
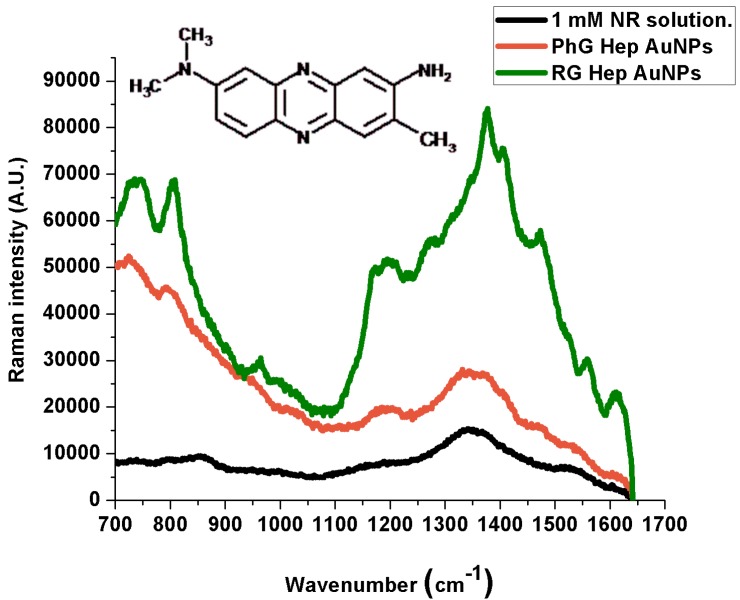
Normal Raman and SERS spectra of Neutral Red.

The Normal Raman and SERS spectra, peak assignment and structure are shown for each dye in Figure 3, Figure 4 and Figure 5. When it comes to metal nanoparticles, spontaneous adsorption occurs if the analyte possesses a charge opposite to that of the particles. Most colloidal metal particles prepared by the reduction of metal salts (in our case, photochemically) carry a negative charge as a result of an adsorbed layer of stabilizing anions, so they will be electrostatically attracted to the nanoparticles surface. If the target molecule carries the same charge as the nanoparticle, it will not interact electrostatically with the metal surface, but still, it can make contact with it by displacing an existing ion from a site on the surface [27]. Table 1, Table 2 and Table 3 indicate the corresponding peak assignments; we determined non-referenced peaks.

**Table 1:** Methylene Blue Raman peak assignment.

Reported Powder Peaks (cm^−1^) [28]	1 mM Aqueous Solution (cm^−1^)	PG-Heparin AuNPs (cm^−1^)	RG-Heparin AuNPs (cm^−1^)	Band Assignment
1067 (w)	1038 (w)	1033 (w)	1037 (m)	C‑H in-plane bending
1121 (w)	-	-	1122 (w)	C‑H out-of-plane-bending
1181 (m)	1185 (w)	1181(w)	1178 (m)	C‑N stretching
1272 (w)	1299 (w)	1293 (w)	1299 (m)	-
1396 (m)	1393 (m)	1386 (m)	1393 (s)	C‑H in-plane ring deformation
1441 (w)	-	-	1464 (w)	C‑N asymmetric stretching
1544 (w)	1541 (w)	1537 (w)	1541 (w)	C‑C ring stretching
1618 (s)	1620 (m)	1607 (m)	1619 (s)	C‑C ring stretching

**Table 2:** Rose Bengal Raman peak assignment.

Reported Peaks (cm^−1^) [29]	1 mM Solution (cm^−1^)	PhG Heparin Au NPs (cm^−1^)	RG Heparin Au NPs (cm^−1^)	Band Assignment
616	-	-	-	-
761	-	787 (w)	793 (w)	C‑Cl stretching
958	-	941 (vw)	947 (w)	-
1012	-	997 (vw)	1000 (w)	C‑OH stretching
1166	-	1170 (vw)	1172 (w)	C‑O and C‑C stretching, C‑H skeletal deformation
1270	-	1280 (vw)	1274 (w)	CCC skeletal deformation in ring, C‑H skeletal deformation
1297	1299 (vw)	-	1291 (w)	CCC skeletal deformation in ring, C‑H skeletal deformation
1340	1343 (m)	1324 (m)	1322 (m)	C‑C stretching in ring
1491	1484 (w)	1478 (vw)	1489 (m)	C=C asymmetric stretching in ring
1553	1551 (vw)	1547 (vw)	1537 (w)	C‑C stretching in ring
1615	1613 (vw)	1603 (w)	1605 (s)	C=C symmetric stretching in ring

**Table 3:** Neutral Red Raman peak assignment.

1 mM Solution (cm^−1^)	PG Heparin Au NPs (cm^−1^)	RG Heparin Au NPs (cm^−1^)	Band Assignment
1605 (w)	1618 (w)	1609 (m)	Scissor vibration of primary amines
1341 (vw)	1336 (w)	1374 (m)	C‑N stretching vibration.
1370 (w)	1370 (w)	1403 (w)	C‑N stretching vibration
1459 (vw)	1470 (w)	1472 (w)	CH_3_ deformation vibration

The SERS spectra show that reactive-grade heparin prepared nanoparticles (RGHep lines) lead to better SERS signals than the pharmaceutical-grade heparin products (PhGHep lines), the only differences arising from the different charges of the analytes, that is, the best interactions were obtained from the oppositely charged one (Methylene Blue) while not being the case with same-charged (Bengal) and neutral (Neutral Red) molecules. Even though some enhancement is observed, which indicates that nanoparticles have made some contact with the metallic surface, it has not been enough to quench such effects [30]; this might be due to the molecule orientation or unbound parts of it.

In the Methylene Blue SERS spectra, the peaks that result from the most enhanced signals are at 1386 and 1617 cm^−1^ (PhGHep AuNPs) and at 1393 and 1619 cm^−1^ (RGHep AuNPs). As for Rose Bengal, such peaks are located at 1324, 1478 and 1603 cm^−1^ (PhGHep AuNPs) and at 1322, 1489 and 1605 cm^−1^ (RGHep AuNPs). Finally, for Neutral Red, although signal enhancement is not very intense, the strongest interactions are indicated by the peaks at 1336 (PhGHep AuNPs) and 1374 cm^−1^ (RGHep AuNPs). According to the assignation in the case of MB, the aromatic rings interact the most with the metal surface, being the same for Rose Bengal. Also, the chloride ions belonging to the dye structure, promote some adhesion to the metallic surface by the displacement of ions they induce in addition to the fact that they might also induce some aggregation on the colloid-dye system, which contributes to the generation of the Rose Bengal SERS signal. Lastly, for Neutral Red, the most acute peak corresponds to the C-N stretching vibration and considering that it possesses a NH_2_ group attached to an aromatic ring and is positively charged, it can be concluded that is the channel through which an interaction, though weak, is established.

When a molecule binds to a metal surface, it can be either physisorbed or chemisorbed. In the case of physisorption, the spectra of physisorbed molecules and free molecules are similar. However, when the molecules are chemisorbed on the metal surface, the position and relative intensities of the SERS bands are dramatically changed due to the overlapping of the molecular and metal orbitals that leads to the formation of a new metal-molecule SERS complex [31]. Here, the probe molecules have been chemisorbed in relation to the 1 mM solution prepared from each dye.

Reactive grade heparin shows better interaction with analytes than the pharmaceutical grade one. This could be due to the higher degree of purity from the first one, with respect to pharmaceutical grade heparin, which is less pure, probably due to the fact that it is administered to humans, so its sulfation level is lower. Also, the acid nature of pharmaceutical grade nanoparticles might promote less contact between the nanoparticles surface and the analytes as will be discussed later.

### 2.3. IR Spectroscopy Measurements

**Figure 6:**
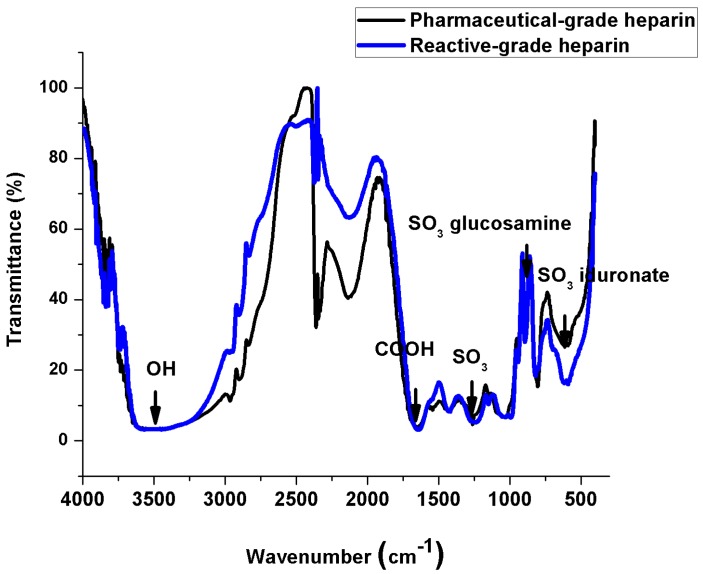
IR spectra of pharmaceutical and reactive-grade.

Figure 6 depicts IR spectra taken from both heparin types, and in general, they exhibit rather comparable features differing only in the sulfate groups content [32,33,34], especially in the glucosamine (941 cm^−1^) and iduronate (800 and 816 and 820 cm^−1^) sections of the spectra, meaning that reactive-grade heparin is more sulfated and has a larger molecular weight. Also, it is possible that the lower sulfation degree in the pharmaceutical-grade heparin might be due to the fact that it is administered to humans, and sulfates, at high concentrations are harmful. Besides this, the reducing power in each heparin is different; the reducing power is found in the glucosamine monosaccharide of heparin and if reactive-grade heparin is bigger and more sulfated, it means that there are more monosaccharides present in its structure, thus promoting more reducing efficiency during the nanoparticle synthesis, yielding a more monodisperse distribution, which is the opposite for pharmaceutical-grade heparin nanoparticles.

Heparin is strongly acidic and negatively charged because of its content of covalently linked sulfate and carboxylic acid groups. In heparin sodium, the acidic protons of the sulfate units are partially replaced by sodium ions, and the reason why heparin is a negatively charged molecule.

### 2.4. Measurements of pH

Measurements of pH were taken in order to find out how the acidity or basicity of the as-prepared colloids, as well as the mix between them and the dyes, affects the SERS signal. The corresponding results are shown in Table 4. The pharmaceutical-grade heparin functionalized gold colloids had a slightly acidic value that in general diminished when the colloids were mixed with the dye analytes, becoming more acidic. On the other hand, for the reactive-grade heparin functionalized gold colloids (unlike the PhGHep AuNPs) the behavior was completely different. The initial pH value (4.86) went up when the dye analytes were added, but still remained in the acidic domain. These results suggest that a very acid environment does not favor SERS signal enhancement, whereas a close to neutral environment benefits it. It can be said that this last condition promotes interactions between nanoparticles and analytes, that are predominantly electrostatic, and allows ion displacement in the case of oppositely charged probe molecules. Another possible explanation for better interactions is that at higher pH values a hydroxyl-rich environment is generated resulting in stronger electrostatic interactions, especially for the positively charged dye. On the contrary, when the media is quite acid, the weak interactions are predominant, most likely of the pi-type, due to the probe molecules being aromatic [35], and result in a protonation state that promotes much less adsorption of the analytes [36].

**Table 4:** pH measurements for nanoparticle syntheses and their mixtures with dyes.

Sample	pH
Pharmaceutical-grade nanoparticle synthesis	6.78
PG Hep NPs-Methylene Blue	6.24
PG Hep NPs-Neutral Red	6.10
PG Hep NPs-Rose Bengal	5.94
Reactive-grade nanoparticle synthesis	4.86
PG Hep NPs-Rose Bengal	5.87
PG Hep NP-Methylene Blue	6.30
PG Hep NPs-Neutral Red	6.60

## 3. Experimental Section

### 3.1. Materials and Methods

Chloroauric Acid (HAuCl_4_) and heparin sodium salt powder (195.9 USP units/mg) were obtained from Sigma-Aldrich. Pharmaceutical grade heparin (5000 IU/mL) was supplied by Pisa Laboratories (Mexico) and benzyl alcohol was from Karal SA de CV. Rose Bengal (Sigma-Aldrich, Saint Louis, MO, USA), Methylene Blue (Industrial KEM, León, Guanajuato, Mexico) and Neutral Red (Hycel de Mexico, SA de CV) were used as the probe molecules. Deionized water was used in all processes. All reactants were used as received.

### 3.2. Nanoparticle Synthesis

The nanoparticles used for SERS experiments were synthesized by preparing a 4 mL solution of either reactive-grade or pharmaceutical-grade heparin at a concentration of 0.833 mM and chloroauric acid at 0.840 mM .The pharmaceutical grade heparin excipient is a mixture of water and benzyl alcohol, so for both experiments to be carried out under the same conditions, such solvent mixture was prepared at the same ratio for dissolving the reactive-grade heparin.

The as-prepared solution was poured in a 10-mm quartz cell to be irradiated for seven hours inside a home-made aluminum cylindrical UV-reactor (the temperature inside varied between 35 and 37 °C), after which the nanoparticles were centrifuged 4 times at 13,500 rpm and redispersed in deionized water. The centrifugation cycle was performed this way due to the fact that heparin is a high molecular weight material that forms a thick shell around nanoparticles which in turn can contribute to SERS signals being weak or not seen at all because there is not enough contact between the gold metal surface and the analyte under study for its detection [37].

### 3.3. Sample Preparation for Characterization

The UV-Vis spectra for colloids and their mixtures with analytes were taken with a UV-Vis Cary 60 spectrometer using 1 mm quartz cuvettes.

SEM images were obtained with a JEOL JSM-7800F Extreme-resolution Analytical Field Emission microscope (JEOL USA Inc., Peabody, MA, USA). The colloids were prepared by washing silicon wafers thoroughly with soap, water and ethanol and then dropping 10 µL of each synthesized solution, *i.e.*, pharmaceutical-grade heparin nanoparticles and reactive-grade prepared ones.

Measurements of pH were obtained using a Conductronic pH120 pH meter (Conductronic, Puebla, Mexico) by pouring solutions in a beaker and dipping the electrode tip to get the corresponding values.

The solutions used for SERS experiments were prepared by mixing 500 µL of colloid and 1 µL of analyte to reach a concentration of 1.996 µm for each analyte. They were incubated for 4 h in the dark after which 200 µL of each mix solution were poured into a polished stainless steel vase using a micropipette to perform measurements. The normal Raman and SERS spectra were acquired with a Renishaw InVia microscope system using the 785 nm laser line (1200 rulings/millimeter) and a 20× objective. The power used was 3 mW and the integration time was 20 s. The presented SERS spectra are the average resulting from measuring different spots on several samples.

## 4. Conclusions 

It was shown that heparin-functionalized gold nanoparticles, obtained using a photochemical reduction, exhibit good Raman activity, which varies according to the type of heparin used as well as to the charge of the analytes under study. Considering that heparin functionalization allows a good affinity for a huge variety of organic molecules and proteins, the results presented here suggest that these heparin-functionalized gold nanoparticles could be utilized in biological applications.
